# Evaluation of a German version of the tonsil and adenoid health status instrument

**DOI:** 10.1186/s40463-014-0041-7

**Published:** 2014-10-31

**Authors:** Teresa Steinbichler, Birte Bender, Elisabeth Blassnigg, Herbert Riechelmann

**Affiliations:** Department of Otorhinolaryngology, Medical University of Innsbruck, Anichstr 35, A-6020 Innsbruck, Austria; Department of Otorhinolaryngology, Community Hospital Kufstein, Endach 27, A-6330 Kufstein, Austria

**Keywords:** Chronic tonsillitis, Quality of life, Cronbach’s alpha, Test-retest reliability, Receiver operating characteristic analysis, Guyatt’s responsiveness Index

## Abstract

**Background:**

To create and validate a German version of the Tonsil and Adenoid Health Status Instrument (TAHSI) for evaluation of tonsillectomy outcome in adult patients with chronic or recurrent tonsillitis.

**Subjects and methods:**

46 healthy volunteers were assessed twice in a 6 week interval with the TAHSI questionnaire. Their results were compared with 45 patients suffering from chronic tonsillitis before tonsillectomy and 6 months following surgery. For internal consistency, Cronbach’s alpha was calculated; to identify normal score values, the optimum cutoff between healthy and diseased individuals was identified with receiver operating characteristic analysis; and responsiveness was assessed using Guyatt’s Responsiveness Index (GRI).

**Results:**

Cronbach’s alpha for all questions was 0.92. Test- retest intraclass correlation coefficient was 0.89 (95% confidence interval 0.80-0.94 p < 0.001). Mean score for the healthy individuals was 7.0 (95% confidence interval 4.2-9.7). The optimum cut off score between healthy and diseased was 20 with a sensitivity of 80% and a specificity of 90% to differentiate controls from tonsillectomy patients.

**Conclusion:**

The TAHSI performed well in this validation tests and is considered a favorable instrument to evaluate the effectiveness of tonsillectomy in adults with chronic or recurrent tonsillitis.

## Background

Tonsillectomy is one of the most frequently performed surgical procedures in ENT. In the United States, more than 500,000 tonsillectomies are performed annually in children younger than 15 years [1]. In Germany, 116,727 tonsillectomies were performed in 2012 [2]. The indications for tonsillectomy vary with age. In children younger than 10 years tonsillar hyperplasia, frequently associated with obstructive sleep apnea, was the most common indication. Chronic or recurrent tonsillitis was the most common indication for tonsillectomy in adolescents and young adults [3]. There is good evidence for the effectiveness of tonsillectomy in children with obstructive sleep apnea syndrome [3] and with chronic or recurrent tonsillitis [4]. However, less data are available in adolescents and adults. To evaluate the benefit of tonsillectomy, validated outcome assessment tools are essential.

A widely used instrument in outcome research is the SF-36 questionnaire available in several languages [5,6]. The SF-36 is a generic tool to measure overall health related quality of life (HRQL). It is frequently used in prospective studies to compare the health related quality of life before and after a medical intervention. The SF-36 was employed to assess tonsillectomy outcome in adults with recurrent tonsillitis in a small group of adult patients [7]. The authors observed a significant improvement in all HRQL scales except mental health 1 year following tonsillectomy. Ericsson and coworkers used the SF-36 to compare radiofrequency-tonsillotomy with conventional tonsillectomy in adolescents and young adults. In both treatment arms, HRQL improved significantly following the intervention, while no significant differences between both groups were found [8]. The Glasgow Benefit Inventory (GBI) is a validated generic instrument for outcome assessment after otorhinolaryngologic interventions. The retrospective, 18-item postintervention questionnaire is available in several languages [9]. The health status prior to the intervention is not assessed. The GBI was used to assess tonsillectomy related change of quality of life in adults with chronic tonsillitis [10-12]. Consistently, tonsillectomy improved general health status in adults with chronic tonsillitis.

Currently, few disease- and intervention-specific outcome assessment instruments are available. In 2006 Baumann and coworkers presented a modified version of the GBI to measure specific symptom responses to tonsillectomy, the SBTI (Specific Benefits from Tonsillectomy Inventory). The authors assessed the 2 disease specific scales ‘symptom change’ and ‘reduced use of resources’ with 5-point Likert scales [13]. Recently, Skevas and coauthors had developed and validated a specific instrument to measure the effectiveness of tonsillectomy, the Tonsillectomy Outcome Inventory 14 in German language. This instrument was validated in a prospective study on 43 patients and showed a good sensitivity and validity but intermediate internal consistency [14]. The Tonsil and Adenoid Health Status Instrument (TAHSI) is a widely used disease specific instrument to determine the outcome of adenotonsillectomy in children. It was developed by Stewart and coworkers in 2001 [15] and proved as a powerful instrument to evaluate the quality of life of children undergoing adenotonsillectomy before and after surgery [16]. The TAHSI includes 6 subscales: airway and breathing, infection, health care utilization, eating and swallowing, cost of care and behavior. Each subscale is assessed with three questions employing 5 point Likert scales. The questionnaire was designed to allow its use in telephone interviews. In 2008 Witsell and colleagues adapted the TAHSI for adults to evaluate the quality of life for adults with chronic tonsillitis after tonsillectomy [17]. In this study, 6 months postoperative results remained stable over 1 year. However, this instrument is not yet systematically validated.

In this study we present a German version of the TAHSI, translated and adapted by two medical experts. It was validated in a prospective test-retest study on 46 healthy adults and a matched number of patients suffering from chronic tonsillitis before and after tonsillectomy.

## Methods

### German TAHSI

The original TAHSI for adults was independently translated into German by two medical experts and finally the two versions were fused. The German version of the TAHSI score (G-TAHSI) consists of 9 subscales: recurrent throat infections, chronic throat infections, eating and swallowing problems, lymphadenopathy, halitosis, health care utilization, severe throat infections, work performance, airway and breathing. Each subscale is covered with 2 questions resulting in 18 questions for the whole questionnaire. Each question is scored with a 5-point Likert scale that assesses the severity of symptoms, ranging from 0 (no problem) to 4 (very severe problem). The points are summed up so that the total score ranges from 0 to 72. High scores indicate a high burden of disease.

### Study participants

A total of 46 healthy adults between 18 and 65 years volunteered in a prospective study between May and June 2013. Volunteering participants were informed about the study and after giving their consent, filled in the TAHSI questionnaire. They were chosen randomly from the hospital staff. Inclusion criteria were good knowledge of the German language, age between 18 and 65, good general health, no chronic or acute throat problems or chronic or recurrent tonsillitis, no tonsillectomy in the past and no malignant disorders. Exclusion criteria were accordingly any kind of chronic or acute throat problems or tonsillitis and adenotonsillectomy or tonsillotomy performed in the childhood. To determine test-retest reliability, all participants filled in the questionnaire again after 6 to 8 weeks, either by personal interview or by telephone interview.

Healthy individuals were chosen as control group to determine the ability of the TAHSI questionnaire to differentiate between healthy individuals and ones suffering from chronic or recurrent tonsillitis.

In a second part of the trial, 46 adult patients with chronic or recurrent tonsillitis presenting at the Department of Otorhinolaryngology – Head & Neck Surgery, Medical University Innsbruck were recruited. All patients were scheduled for tonsillectomy. They were questioned to participate in a clinical trial. For this trial, a positive vote of the ethical review board of the Medical University Innsbruck was obtained (UN3796 / 282/4.7). Patients were prospectively interviewed before they underwent tonsillectomy and 6 months after surgery with the TAHSI questionnaire. Inclusion criteria for this part of the trial were diagnosis of chronic or recurrent tonsillitis and age between 18 and 65. Recurrent tonsillitis in adults was defined as 3 or more documented episodes of acute tonsillitis per year requiring antibiotic treatment in the last two years. The diagnosis of chronic tonsillitis was also made in patients with sore throat for at least three months, accompanied by tonsillar inflammation, despite appropriate antibiotic treatment [18]. Exclusion criteria included abscess tonsillectomy, tonsillectomy for proven or suspected neoplasia and mere tonsil hyperplasia without inflammation.

### Data analysis

Mean and standard deviations are provided for score values, categorical data are presented as frequency tables. Some results were graphically displayed with box plots. Internal consistency of the 18 questionnaire items was assessed with Cronbachs α in the healthy volunteers group. Receiver operating characteristic (ROC) analysis was used to estimate an optimum cutoff score between healthy and diseased using the data of both groups. As a measure of responsiveness, Guyatt’s Responsiveness Index [19] was calculated in the patients group before and 6–8 months following tonsillectomy. All data were analyzed using SPSS Statistic Vers. 21 (IBM Corporation, Armonk, NY).

## Results

In the control group, 46 healthy persons met the inclusion criteria and were enrolled. Of these 46 volunteers, 32 (70%) were female; the mean age was 30 years. The mean TAHSI-score was 6.9+/−9.3 (mean+/− SD) at the first and 5.9+/−8.7 at the second interview, which makes approximately 10% of the maximum achievable score. The 95% confidence interval for the TAHSI-score in healthy volunteers was 4.2 to 9.7. Cronbach’s alpha for all 18 questions was 0.92 for the first interview and 0.93 for the second.

In the tonsillectomy group, also 46 patients were enrolled. Sex distribution was similar to the control group, 78% of the patients in the tonsillectomy group were female. On average, patients in the tonsillectomy group were younger than the controls. The mean age in the patient group was 26.4+/−7.6 years. In the tonsillectomy group, 1 patient was lost to follow up and excluded. All subscales were filled in by all participants.

**Figure 1:**
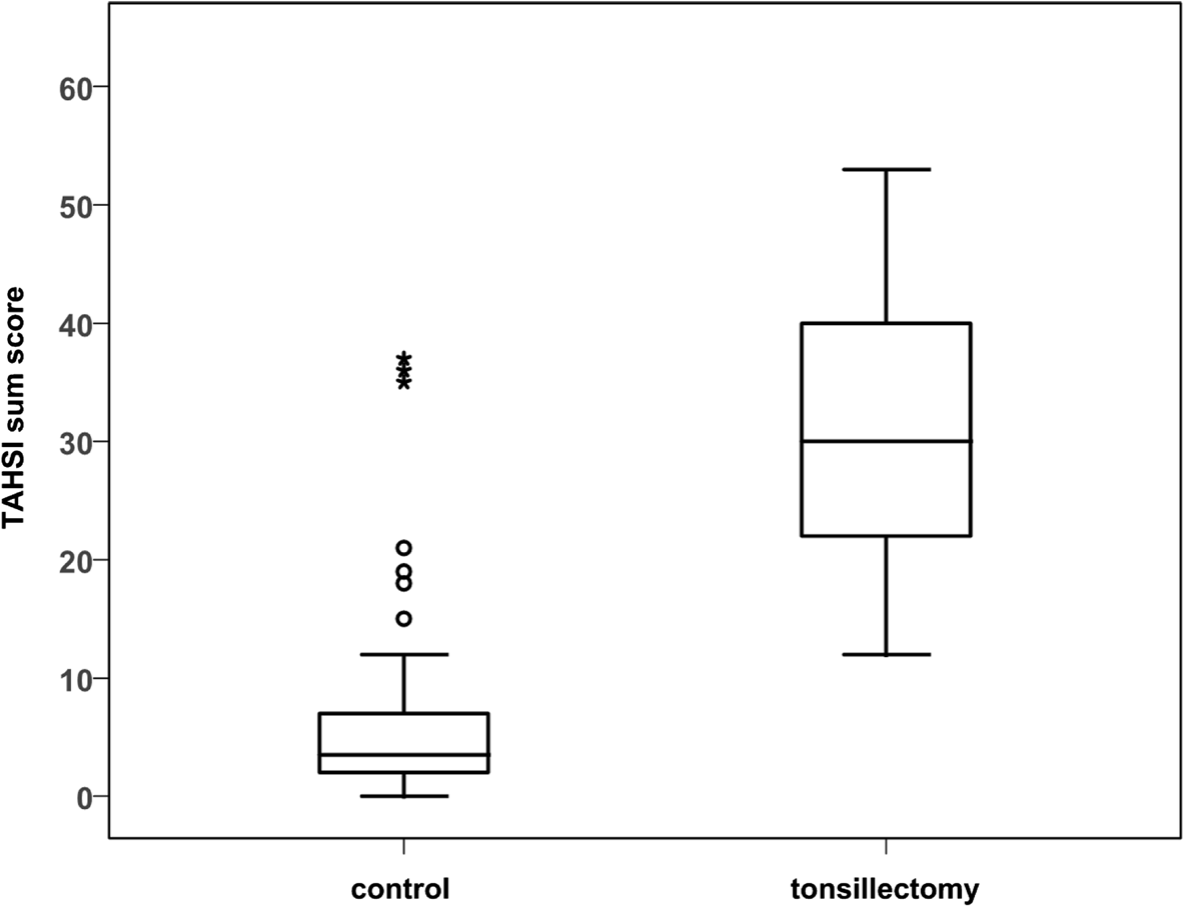
**Box and whiskers plot illustrating TAHSI scores of healthy controls and scores of patients: box and whiskers plot illustrating TAHSI scores of healthy controls (n = 46) compared with the scores of patients planned for tonsillectomy for chronic tonsillitis (n = 45).** Bar: median; boxes: quartiles; whiskers: Tukey hinges; circles: outliers; asterisks: far outliers; Mann–Whitney test: p < 0.001).

**Figure 2:**
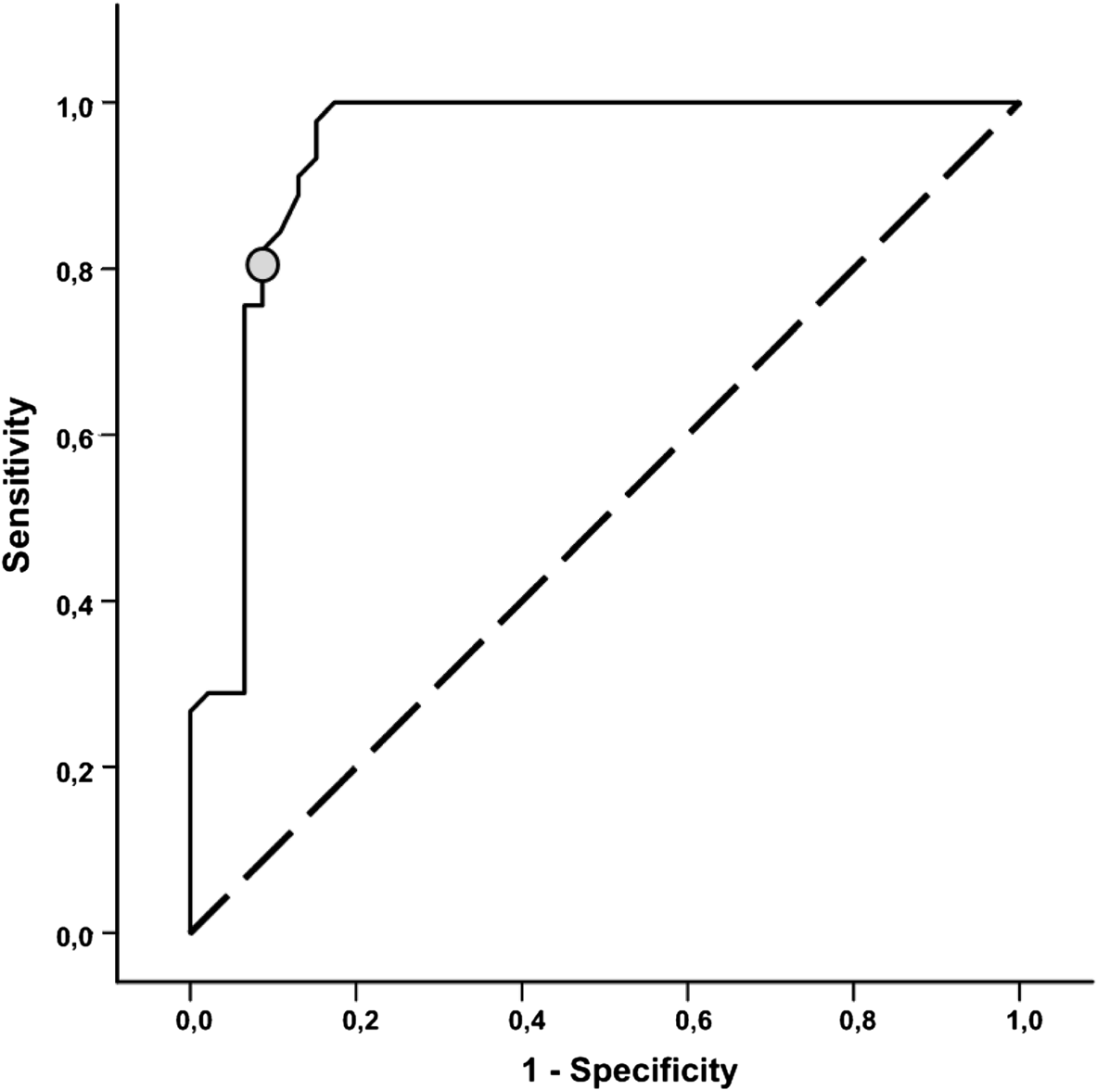
**Receiver operating characteristic (ROC) curve: receiver operating characteristic (ROC) curve illustrating the true positive rate (sensitivity; y-axis) vs. the false positive rate (1-specificity; x-axis) at increasing TAHSI-score values.** The area under the ROC curve was 0.94 (95% CI 0.89 to 0.99; p < 0.001). At a cut-off score of 20 (circle), sensitivity to detect members of the tonsillectomy group with chronic or recurrent tonsillitis was 80%, the specificity 90%.

The mean TAHSI-score in the tonsillectomy group before surgery was 31.1+/−11.3, which makes approximately 40% of the maximum achievable score. The mean scores between controls and patients before tonsillectomy differed significantly (p < 0.001) (Figure 1). The power of the TAHSI score to discriminate healthy individuals from patients with chronic or recurrent tonsillitis scheduled for tonsillectomy was assessed by ROC analysis. The area under the ROC curve was 0.94 (95% CI 0.89 to 0.99; p < 0.001). At a cut-off score of 20, sensitivity to detect members of the tonsillectomy group with chronic or recurrent tonsillitis was 80%, the specificity 90% (Figure 2). All 9 subscales of the TAHSI were significantly higher in the tonsillectomy group when compared with the controls. Data for nocturnal breathing were not available in the tonsillectomy group. Health care utilization scored highest in the tonsillectomy group.

**Figure 3:**
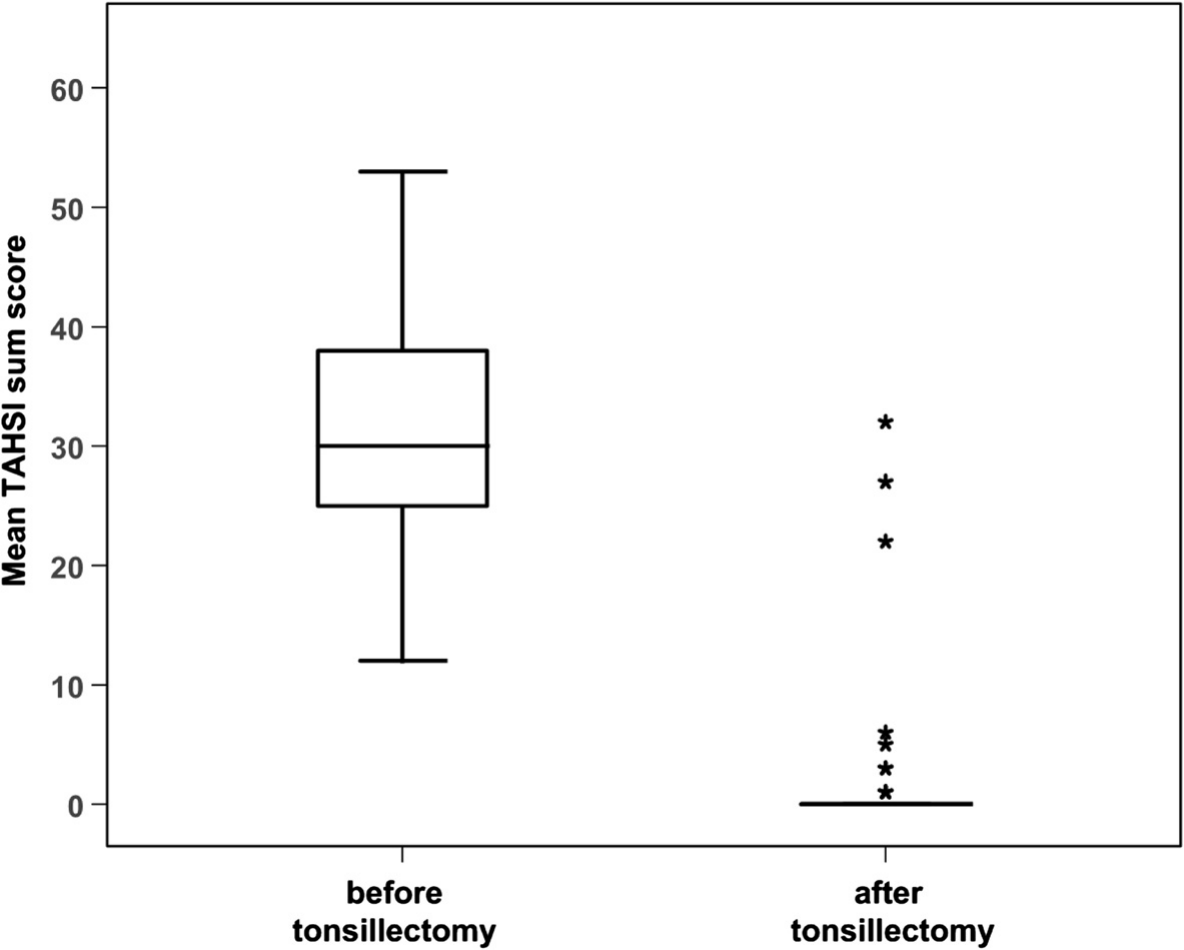
**Box and whiskers plot illustrating TAHSI scores following tonsillectomy: box and whiskers plot illustrating TAHSI scores of patients with chronic tonsillitis (n = 40) before and 6 months following tonsillectomy.** (Wilcoxon signed rank test p < 0.001).

Six months following surgery, mean TAHSI score in the tonsillectomy group had dropped from 31.1+/−11.3 to 7.0+/−9.3 (p < 0.001) (Figure 3). Assuming a cut- off of 20 score points between normal and diseased, all patients except 3 (approximately 95%) achieved a score below 20.6 months following surgery. To assess responsiveness of the TAHSI, Guyatt’s Responsiveness Index was calculated. For this index, the ratio of the mean change of patients in the treatment group was divided by the standard deviation of change in the control group. Guyatt’s Responsiveness Index for the TAHSI questionnaire was 5.2.

## Discussion

Several generic instruments to assess tonsillectomy outcome are currently available. In a recent review, Andreou and coworkers reported that the SF- 36 and the Glasgow Benefit Inventory are most frequently used [20]. The SF-36 is a universal HRQL assessment tool with well documented psychometric characteristics [6]. The Glasgow Benefit Inventory is a generic instrument adapted to otorhinolaryngological procedures. Its main limitation is that pre- and postoperative states are not assessed separately, but subjective change parameters are retrospectively measured [9].

However, only few disease- and intervention-specific outcome assessment instruments for tonsillectomy are available. The Tonsil and Adenoid Health Status Instrument (TAHSI) for use in children with tonsil and adenoid disease was initially developed to assess the effect of adenotonsillectomy on disease specific quality of life in affected children [15]. As its name says, the TAHSI was constructed to measure tonsillar disease specific individual health status. Personal health status is an individual’s relative level of wellness and illness, taking into account the presence of biological or physiological dysfunction, symptoms, and functional impairment, standardized for the typical patient [21]. In contrast to HRQL, it does not primarily reflect the level of happiness and satisfaction related to health status. The subscales of the original TAHSI for children were developed using principal component analysis. The authors identified 6 subscales including eating and swallowing, airway and breathing, infections, health care utilization, cost of care, and behavior. The authors used one-dimensional 5 point Likert scales with high scores always indicating a poor health status. Psychometric characteristics of this instrument have been systematically evaluated and revealed a favorable profile. The public domain instrument is suitable for prospective studies, because the tonsil-related health status can be assessed before and after the intervention.

In 2008, Witsell and coworkers adapted the TAHSI to adolescents and adults [17] using the same subscales. Although not yet formally validated, this instrument seemed suitable for translation in other languages. In the translated form presented here, the infection subscale was further differentiated into recurrent throat infections, chronic throat infections, severe throat infections and lymphadenopathy. Moreover, a subscale for halitosis and work or school performance was added. Finally, the translated version consisted of 9 subscales with 2 questions each resulting in 18 questions (Table 1).

**Table 1:** **Nine subscales of the German version of TASHI**

**Subscale**	**Questions**
Recurrent throat infections	My throat was aching frequently for a few days.
	I often had a sore throat for a few days.
Halitosis	I often had bad breath or a bad taste in my mouth.
	I was worried about having bad breath.
Chronic throat infection	My throat was aching continuously.
	I always had at least a slightly sore throat.
Swallowing problems	I had problems swallowing.
	Swallowing caused difficulties.
Lymphadenopathy	I had a painful swelling on the neck as well.
	I had swollen lymph nodes on the neck.
Health care utilization	I had to consult a doctor because of my throat problems.
	Due to my throat problems I had to buy medicine from the pharmacy.
Severe throat infections	I had fever because of my throat inflammation.
	Because of throat inflammations I had to stay in bed.
Work performance	Due to my sore throat my performance in school and work suffered.
	My throat issues limited my capability in school and work.
Nocturnal breathing	My breathing was very noisy during the night.
	I snored loudly.

In the right columns, the 2 questions per subscale were retranslated from German to English.

In this validation trial we used healthy individuals as a control group, because we wanted to determine the power of the TAHSI score to discriminate between healthy and diseased individuals. We did not assess the effectiveness of tonsillectomy by comparing the disease related quality of life after tonsillectomy with the one after conservative treatment for the same time.

This translated form was formally validated employing common criteria [22]. Reliability was assessed using Cronbach’s alpha. Cronbach’s alpha is a widely used measure of internal consistency. For the translated version of the TAHSI for adults, Cronbach’s alpha was 0.92, which lies within the acceptable range for monitoring of individual scores [22]. For comparison, a Cronbach’s alpha of 0.75 to 0.85 was calculated for the SF-36 in 1876 patients [23]. Assessment of test-retest reliability demonstrated the stability over time of the TAHSI score. The reliability coefficient was 0.89. In general a value over 0.8 is adequate. For comparison, the SF-36 has a reliability coefficient over 0.75 in all dimensions except social functioning. Generally, reliability coefficients of 0.90 or more are considered adequate for individual measurement over time according to the Scientific Advisory Committee of the Medical Outcomes Trust [22].

The high scores found in patients diagnosed with chronic or recurrent tonsillitis by medical specialists in our outpatient clinic and the low scores in controls suggests appropriate construct validity of the TAHSI. Responsiveness is another important part of the longitudinal construct validation. It was determined with the Guyatts Responsiveness Index. The GRI measures changes brought by an intervention in relationship with random changes. The GRI for the TAHSI was 5.1. This indicates high sensitivity to changes in tonsil related health status. A GRI over 0.8 is considered to reflect good responsiveness [24-26].

**Figure 4:**
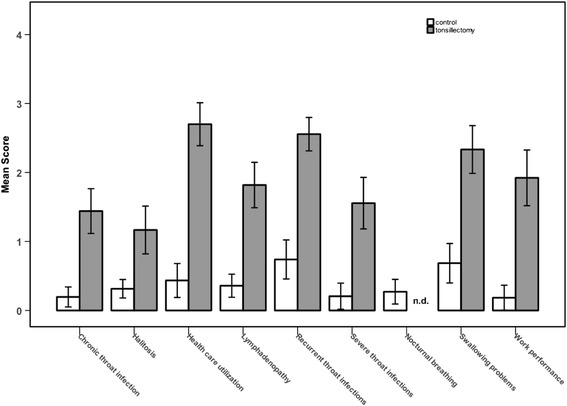
**Mean scores (minimum 0; maximum 4) of the 9 subscales of the modified TAHSI questionnaire: the scores of the 2 questions of each subscale per participant were averaged and then the mean of the sample was calculated.** White bars: control group; grey bars: patients scheduled for tonsillectomy Error bars represent 95% confidence intervals (n.d. = no data).

Criterion related validity was demonstrated with the help of receiver operating characteristic [22]. The TAHSI had a sensitivity of 80% and a specificity of 90% at a cut-off at 20, so that the indication for tonsillectomy may be made conservatively using a cut-off at 20 score points. The TAHSI score was easy to understand for the patients and filled out completely by all study participants. Consistently, a high score means a high burden of disease. All subscales revealed significantly higher scores in the tonsillectomy group (Figure 4). The additional stratification into recurrent and chronic throat infection and the additional subscales halitosis and work performance allowed identification of the patient subsets by cluster analysis (data not shown). Moreover, it allowed a more detailed description of the individual symptom profile.

Most items of the questionnaire are considered adaptable to other languages and regions of the world. Most subscales except health care utilization deal with patient’s throat symptoms, which are considered more or less intercultural. However, questions on health care utilization, i.e. visiting doctors because of throat problems or access to throat medications may depend on cultural conventions and national health care regulations. Moreover, questions on work performance may require adaptation, for instance in mainly rural areas. It is also unclear, if bed rest is a common strategy to cope with severe throat infections in different regions of the world. The whole questionnaire was set in plain words. In most cases, the participants could fill out the questionnaire without assistance. The time effort was approximately 2 minutes. The TAHSI is freely available and can reliably be assessed by telephone interview [4,27].

## Conclusion

In summary this study delivers a reliable assessment tool for physicians to estimate the effectiveness of tonsillectomy for people with chronic tonsillitis. High TAHSI scores may also aid in the clinical decision for tonsillectomy.
